# Obesity in the GLP-1 Era: Clinical Characteristics and Prescribing Patterns in a Large Integrated Health System

**DOI:** 10.64898/2026.09.04.26361064

**Published:** 2026-09-08

**Authors:** Brian J Wells, Kristin M Lenoir, Michael Webster-Clark, Nicholas M Pajewski, Amanda T Pons-Junkins, Michelle M Mielke

## Abstract

**Aims:**

To characterize adults with obesity in a large integrated health system.

**Methods:**

We created a cross-sectional cohort of adults with at least one body mass index (BMI) value >=30 kg/m2 in the prior 18 months, a recent in-person outpatient visit, and an assigned primary care provider. Electronic health record data were transformed into a patient-level analytic dataset and linked to neighborhood-level social determinants of health. Diagnoses, laboratory values, vital signs, and prescriptions were curated using reproducible phenotype definitions and internally reviewed vocabularies.

**Results:**

The registry included 280,186 adults, of whom 48% had class I obesity. The cohort was 63% female, 27% Black, and 14% lived in areas with high or very high deprivation. Common comorbidities included hypertension, type 2 diabetes, depression, and anxiety. HbA1c and cholesterol measurements were absent in more than 40% of the cohort during the prior two years. Overall, 22% of patients received a prescription for at least one weight loss drug in the past 2 years, including 18% who received a weight-loss-approved GLP-1 receptor agonist.

**Conclusions:**

This care-engaged obesity cohort was characterized by high representation of women and socially vulnerable populations, substantial cardiometabolic screening gaps, and broad adoption of GLP-1 agonists.

## Full Text Availability

The license terms selected by the author(s) for this preprint version do not permit archiving in PMC. The full text is available from the preprint server.

